# miR-26a-5p protects against myocardial ischemia/reperfusion injury by regulating the PTEN/PI3K/AKT signaling pathway

**DOI:** 10.1590/1414-431X20199106

**Published:** 2020-01-24

**Authors:** Xiaowei Xing, Shuang Guo, Guanghao Zhang, Yusheng Liu, Shaojie Bi, Xin Wang, Qinghua Lu

**Affiliations:** 1 Department of Cardiology, The Second Hospital of Shandong University, Jinan, Shandong, China; 2 Department of Gastroenterology, The Second Hospital of Shandong University, Jinan, Shandong, China

**Keywords:** Acute myocardial infarction, miR-26a-5p, PTEN, Apoptosis, PI3K/AKT pathway, Myocardial ischemia/reperfusion injury

## Abstract

Reperfusion strategies in acute myocardial infarction (AMI) can cause a series of additional clinical damage, defined as myocardial ischemia/reperfusion (I/R) injury, and thus there is a need for effective therapeutic methods to attenuate I/R injury. miR-26a-5p has been proven to be an essential regulator for biological processes in different cell types. Nevertheless, the role of miR-26a-5p in myocardial I/R injury has not yet been reported. We established an I/R injury model *in vitro* and *in vivo*. *In vitro*, we used cardiomyocytes to simulate I/R injury using hypoxia/reoxygenation (H/R) assay. *In vivo*, we used C57BL/6 mice to construct I/R injury model. The infarct area was examined by TTC staining. The level of miR-26a-5p and PTEN was determined by bioinformatics methods, qRT-PCR, and western blot. In addition, the viability and apoptosis of cardiomyocytes were separately detected by MTT and flow cytometry. The targeting relationship between miR-26a-5p and PTEN was analyzed by the TargetScan website and luciferase reporter assay. I/R and H/R treatment induced myocardial tissue injury and cardiomyocyte apoptosis, respectively. The results showed that miR-26a-5p was down-regulated in myocardial I/R injury. PTEN was found to be a direct target of miR-26a-5p. Furthermore, miR-26a-5p effectively improved viability and inhibited apoptosis in cardiomyocytes upon I/R injury by inhibiting PTEN expression to activate the PI3K/AKT signaling pathway. miR-26a-5p could protect cardiomyocytes against I/R injury by regulating the PTEN/PI3K/AKT pathway, which offers a potential approach for myocardial I/R injury treatment.

## Introduction

Acute myocardial infarction (AMI) is a common type of ischemic heart disease, with serious consequences in morbidity and mortality (1). Reperfusion therapy that promotes the rapid return of blood flow to the ischemic zone in the myocardium is the major procedure for the treatment of AMI (2). Although reperfusion can ameliorate myocardial function, limit infarct size, and timely and effectively reduce mortality (3), it causes extra damage to the myocardium, a phenomenon called myocardial ischemia/reperfusion (I/R) injury (4). The features of myocardial I/R injury mainly include cardiomyocyte death, microvascular destruction, and inflammation (3,5), which may induce contractile dysfunction, cardiac arrhythmias, and myocardial infarction (6). At present, there is no effective therapeutic method for myocardial I/R injury (7). Hence, it is of great clinical significance to explore the molecular mechanism of the pathological processes of myocardial I/R injury.

MicroRNAs (miRNAs), as small noncoding RNA molecules, mediate gene expression by being complementary to a site in the 3′ UTRs (untranslated regions) of target mRNAs, thereby resulting in mRNA degradation or translational repression (8). miRNAs are linked to many developmental and physiologic processes, mainly including cell metastasis, proliferation, apoptosis and hematopoiesis (9,10). Literature suggests that the abnormal expression level of miRNAs is relevant to the pathogenesis of various diseases (11,12). For instance, miR-29 is critical for regulating tissue fibrosis, which can be regarded as an intriguing therapeutic target for fibrosis diseases (13). Down-regulation of miR-146a has been reported to protect against cardiac hypertrophy and dysfunction induced by high pressure (14). miR-26a-5p acts as either a tumor promoter or suppressor in the development and progression of diverse cancers (15,16). Furthermore, miR-26a-5p has been found to be abnormally expressed in several cardiovascular diseases such as AMI (17,18). However, the function of miR-26a-5p in myocardial I/R injury has not been reported.

Therefore, we established an I/R injury model *in vitro* and *in vivo*. Then, we determined the expression and role of miR-26a-5p in myocardial I/R injury using bioinformatics methods and biological experiments.

## Material and Methods

### Database and bioinformatics analysis

Gene Expression Omnibus (GEO, https://www.ncbi.nlm.nih.gov/geo/) was searched for “heart ischemia reperfusion” and “miRNA” to identify the dataset of myocardial I/R injury-related miRNAs. Datasets with a sample size of less than 10 were excluded. Furthermore, the expression profiles of miRNAs were determined only in samples that suffered myocardial I/R injury, which was used as a key condition for selecting the dataset. Therefore, the dataset of GSE74951 deposited by Feng et al. (19) was chosen, and this dataset based on the platform of the GPL21136 includes nine myocardial I/R injury mice and six sham mice. The data collection processes were in accordance with all regulations. The raw data of miR-26a-5p were analyzed and normalized by the Limma package in the R language (version 3.4.3). The expression difference of miR-26a-5p between myocardial I/R injury mice and sham mice was calculated by Student's *t*-test.

In addition, the potential target of miR-26a-5p was predicted using the TargetScan website (http://www.targetscan.org/vert_72/), and PTEN, a tumor suppressor, was selected as a candidate.

### Cell isolation and culture

The present work was approved by the Ethics Committee of the Second Hospital of Shandong University. All procedures were guided by the Care and Use of Laboratory Animals guidelines from the National Institutes of Health. We purchased C57BL/6 mice from Shanghai Experimental Animal Center of Chinese Academy of Sciences (China). Primary cardiomyocytes were isolated from C57BL/6 mice as previously reported (20). Briefly, ventricles were isolated from 2-day-old mice embryonic hearts, minced, and digested with 0.25% trypsin (Gibco, USA) and 0.2% collagenase type II (Sigma, USA) for two cycles. The extract was centrifuged at 45 *g* at room temperature for 5 min, and the supernatant was removed, followed by the addition of D-Hanks solution (Sigma). The sample was centrifuged at 100 *g* at room temperature for 5 min, the supernatant was removed, followed by adding the culture medium to obtain cell suspension. The obtained cells were seeded and incubated in DMEM with 1% penicillin/streptomycin, 10% fetal bovine serum (FBS) and 4 mM L-glutamine in an incubator with 5% CO_2_ at 37°C.

### Cell transfection

miR-26a-5p inhibitor, miR-26a-5p mimic, and controls (inhibitor control, mimic control) were synthesized from Thermo Fisher Scientific (USA) and Shanghai GenePharma Co., Ltd. (China). To knock down or overexpress miR-26a-5p, primary cardiomyocytes were transfected with miR-26a-5p inhibitor or mimic by Lipofectamine 3000 (Invitrogen, USA).

### Establishment of cardiomyocyte hypoxia/reoxygenation model

To simulate I/R injury *in vitro*, the transfected cardiomyocytes were submitted to hypoxia/reoxygenation (H/R) treatment. The transfected cardiomyocytes were incubated to approximately 80% confluence. Then, DMEM medium was removed and changed with sugar-free and serum-free medium. Next, the cells were cultured in a hypoxic incubator with the atmosphere of 5% CO_2_ and 95% N_2_ for 4 h to simulate ischemia. Subsequently, the cells were transferred to normal DMEM containing 1% penicillin/streptomycin, 10% FBS, and 4 mM L-glutamine in an atmosphere of 95% air and 5% CO_2_ for 2 h to simulate reperfusion. Thus, the H/R model was successfully constructed.

### Establishment of myocardial I/R model

A total of 32 C57BL/6 mice (8 weeks old, 23–25 g) were housed in a constant temperature room (22°C, 12 h light/dark cycle, 50–55% humidity) with water and food *ad libitum*. The mice were randomly divided into two groups (sham group and I/R injury group, 16 per group). After adapting to the environment for 5 days, the mice were anesthetized with 1% pentobarbital sodium (50 mg/kg) intraperitoneal injections before endotracheal intubation. Next, the mice were placed on the operating table in a supine position, had the hair of the neck and chest removed, and were disinfected with iodophor. The limbs were fixed on electrodes to connect to the electrocardiograph (BeneHeart R3, Mindray, China) and a rodent ventilator (R407, RWD Life Science, China) was inserted into the mice mouths, which could monitor the limb lead II electrocardiogram (ECG) with the following conditions: 90–105 times/min respiratory rate, room air supply, and 0.4–0.8 mL tidal volume. Between the third and fourth ribs, left lateral thoracotomy was carried out. Subsequently, the left anterior descending coronary artery (LADCA) was ligated using 8–0 atraumatic suture to produce occlusions. When the notable upgrade of ST segment in ECG was recorded by the ECG monitor, the coronary artery was successfully blocked. The suture was released after 30 min of the block and the heart was reperfused, causing an obvious decline in the ST segment. Thus, the myocardial I/R model was successfully constructed. Next, pneumothorax was evacuated manually, and the chest and skin were closed using a 6-0 Prolene suture. For analgesia, buprenorphine (0.1 mg/kg body weight) was administered *ip*. Henceforth, the mice were kept in clean cages at room temperature. Additionally, the same procedure except the ligation of LADCA was performed on the sham group of mice.

### Determination of myocardial infarct size

Twenty-four hours after reperfusion, we occluded the LADCA again, and injected 0.2 mL of 1% (wt/vol) Evans blue into the aorta. The heart was immediately excised, stored at -80°C, cut into 1-mm-thick slices, and counterstained with 1% (wt/vol) TTC (2,3,5-triphenyltetrazolium chloride; Sigma) solution at 37°C for 15 min. The sample pictures were captured. Then, the infarct area was assessed using Image-Pro Plus 6.0 software. Myocardial infarct size is reported as (infarct area/whole heart area) × 100%.

### Quantitative real-time PCR (qRT-PCR)

Total RNAs from myocardial tissues or cardiomyocytes were extracted using Trizol (Invitrogen). cDNAs were derived from RNA reverse transcription with a test kit (QuantiTect Reverse Transcription Kit, Qiagen, USA). PCR was conducted with Applied Biosystems^®^ ViiA™ 7 Real-Time PCR System (Life Technologies, USA) using SYBRTM Green PCR Kit (Life Technologies). GAPDH was defined as the internal reference. The 2^−ΔΔCt^ method was applied to analyze the relative mRNA expression. The following primers were used: miR-26a-5p forward: 5′‐UCC AUA AAG UAG GAA ACA CUA CA‐3′, reverse: 5′‐CAG UAC UUU UGU GUA GUA CAA‐3′; PTEN forward: 5′-AAG ACC ATA ACC CAC CAC AGC-3′, reverse: 5′-ACC AGT TCG TCC CTT TCC AG-3′; GAPDH forward 5′-GAC TCA TGA CCA CAG TCC ATG C-3′, reverse 5′-AGA GGC AGG GAT GAT GTT CTG-3′.

### Western blot

Total protein from myocardial tissues or cardiomyocytes was lysed using lysis buffer on ice. After quantitation of protein using the BCA method, an equal amount of protein (20 µg) was separated onto 10% SDS-PAGE gels, followed by transferring to PVDF membranes (Millipore, USA) by electroelution. Subsequently, 5% skim milk was utilized to block the membranes for 2 h at 37°C. Next, the membranes were cultured with primary antibodies (dilution 1:1000, Cell Signaling Technology, USA) against PTEN, PI3K, AKT, and cleaved caspase 3 at 4°C overnight, followed by incubating with secondary antibodies conjugated with HRP for 2 h at 37°C. After washing with TBST solution, the protein bands were measured by an enhanced chemiluminescence detection system (Thermo Fisher Scientific). The internal control was actin.

### MTT assay

Forty-eight hours post-transfection, the transfected cardiomyocytes submitted to H/R treatment were seeded onto 96-well plates (Corning Life Science, USA), followed by incubation at 37°C for 24, 48, 72, and 96 h. MTT (5 mg/mL) solution was added to the culture medium and incubated for another 4 h. Subsequently, dimethyl sulfoxide was applied to dissolve the formazan crystals, and absorbance at 490 nm was determined by a microplate reader (BioTek, USA).

### Flow cytometry

Flow cytometry with an Annexin V/PI kit (BD Bioscience, USA) was utilized to detect cell apoptosis. Briefly, cardiomyocytes were harvested, washed with PBS, followed by re-suspending in 1× binding buffer. Next, the cardiomyocytes were stained for 30 min in a darkroom using Annexin V and PI, and FACS analysis was performed with FACSCalibur (BD Biosciences).

### Luciferase reporter assay

The 3′UTR fragments of PTEN containing both mutant (MUT) and wild-type (WT) binding sites of miR-26a-5p were amplified by PCR and cloned into the vector pMIR-REPOR™ Luciferase (Promega Corp., USA) to form luciferase reporter vectors. The vectors and miR-26a-5p mimic were co-transfected into primary cardiomyocytes via Lipofectamine 3000 (Invitrogen). Luciferase activity after 24 h of transfection was determined by a dual luciferase reporter assay kit (Promega) on a Fluoroskan Ascent Type 379 fluorescence plate reader (Thermo), and normalized to renilla luciferase intensity.

### Statistical analysis

Data are reported as means ± SD and were assessed by GraphPad Prism Software (version 7, USA). Each experiment was performed repeatedly at least three times. The difference between two groups was evaluated by Student's *t*-test, and the comparison between more than two groups was analyzed by one-way ANOVA with Bonferroni's multiple comparisons test. A value of P<0.05 was regarded as a significant difference.

## Results

### Establishment of I/R injury model

**Figure 1:**
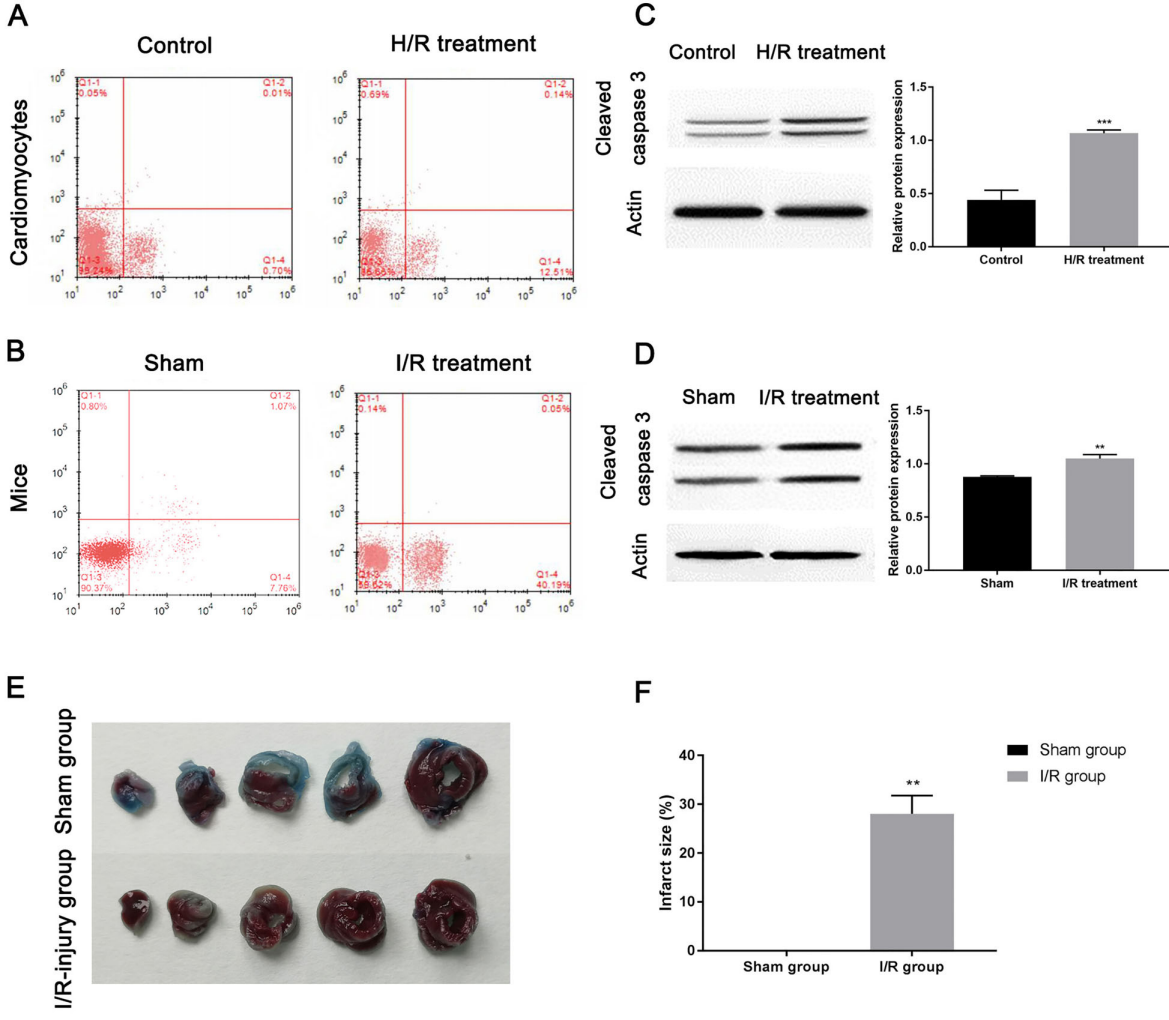
Establishment of ischemia/reperfusion (I/R) injury model. The images of flow cytometry show apoptosis in (**A**) cardiomyocytes and (**B**) myocardium of mice upon I/R injury. Western blot examined the expression of cleaved caspase 3 in (**C**) cardiomyocytes submitted to hypoxia/reoxygenation (H/R) treatment and (**D**) myocardial tissue upon I/R treatment. **E**, Representative images of Evans blue/TTC staining in five continuous slices of left ventricle from mice hearts treated with or without I/R treatment. **F**, The infarct size was quantified by Image-Pro Plus software. Data are reported as means±SD. **P<0.01, ***P<0.001 *vs* control groups (*t*-test).

As cell apoptosis is reported as a major pathophysiological mechanism in myocardial I/R injury (21), flow cytometry was utilized to determine the cardiomyocyte apoptosis in the I/R injury model. Figure 1A revealed that the apoptosis rate of primary cardiomyocytes under H/R treatment was 12.65%, and the apoptosis rate in the control group was 0.71%. In addition, the apoptosis rate of cardiomyocytes after I/R treatment was 40.24% and in the sham group it was 8.83% (Figure 1B). This result was validated by pro-apoptotic protein cleaved caspase 3 expression. Western blot indicated that cleaved caspase 3 level in cardiomyocytes (Figure 1C, P=0.0003) and myocardial tissue (Figure 1D, P=0.0015) upon I/R injury was up-regulated compared to the control groups. Furthermore, Figure 1E and F illustrated that there was no infarct damage in the sham group, and yet the infarct size of the myocardium in the I/R injury model was larger than that in the sham group (P=0.0058). Collectively, the I/R injury model *in vitro* and *in vivo* had been successfully established, and H/R and I/R treatment significantly induced cardiomyocyte apoptosis.

### Expression of miR-26a-5p in I/R-injury mice and H/R-induced cardiomyocytes

**Figure 2:**
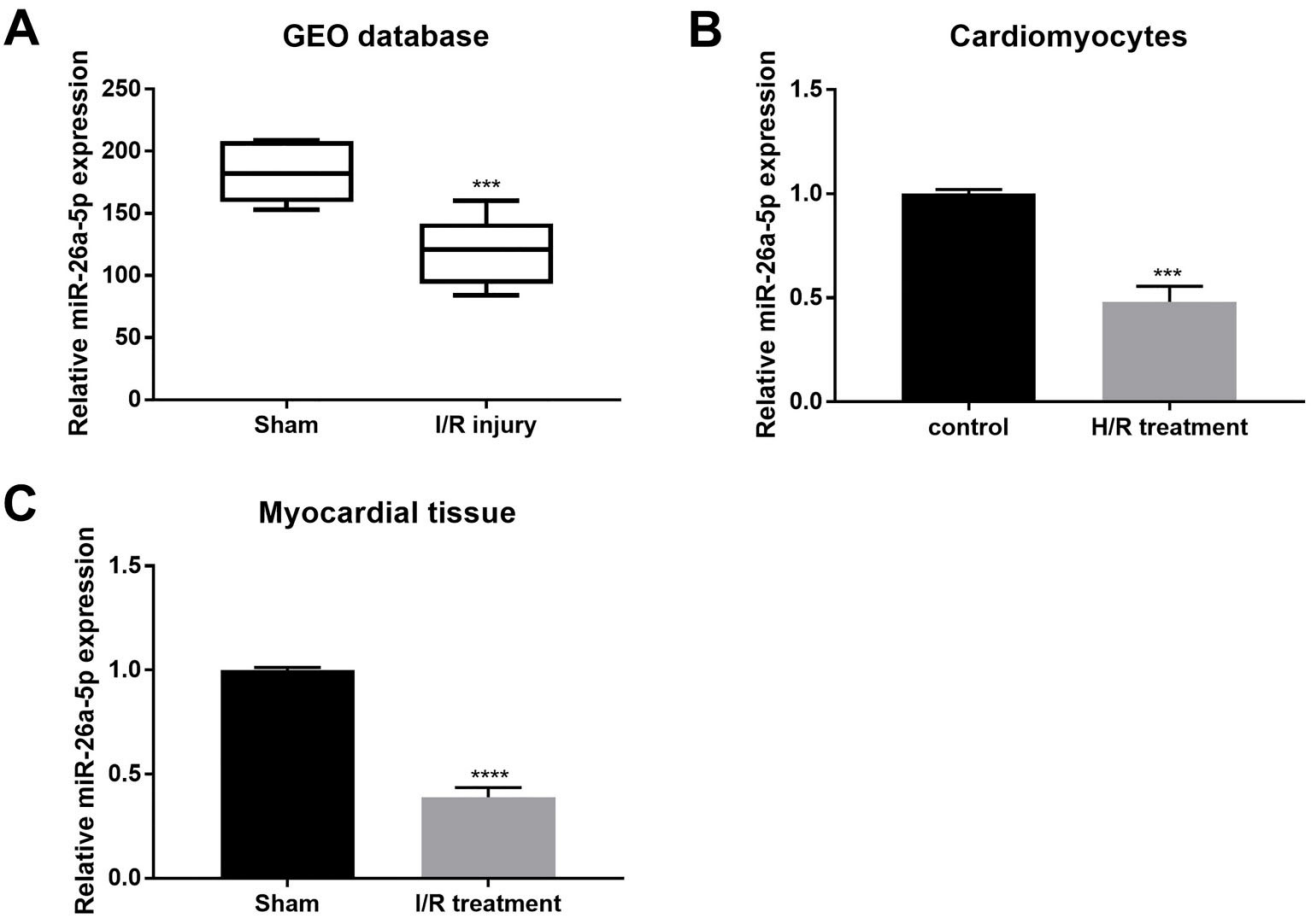
Detection of miR-26a-5p expression. **A**, The expression data of miR-26a-5p from GEO database were analyzed (medians and interquartile ranges). qRT-PCR was applied to analyze the expression of miR-26a-5p in (**B**) cardiomyocytes submitted to hypoxia/reoxygenation (H/R) treatment and (**C**) myocardial tissue upon ischemia/reperfusion (I/R) injury. Data are reported as means±SD. ***P<0.001, ****P<0.0001 *vs* control groups (*t*-test).

To detect miR-26a-5p expression, we applied bioinformatics analysis and qRT-PCR. Firstly, the bioinformatics analysis of miR-26a-5p data from GEO database revealed that its expression was significantly decreased in myocardial I/R injury mice (Figure 2A, P=0.0004). qRT-PCR indicated that miR-26a-5p expression was considerably down-regulated in primary cardiomyocytes with H/R treatment compared to the control group (Figure 2B, P=0.0003). Similarly, miR-26a-5p expression showed a reduction in myocardial tissues of C57BL/6 mice after I/R treatment compared to the sham group (Figure 2C, P<0.0001). Collectively, the expression level of miR-26a-5p was related to I/R injury.

### Effect of miR-26a-5p on cell viability and apoptosis in H/R-induced cardiomyocytes

**Figure 3:**
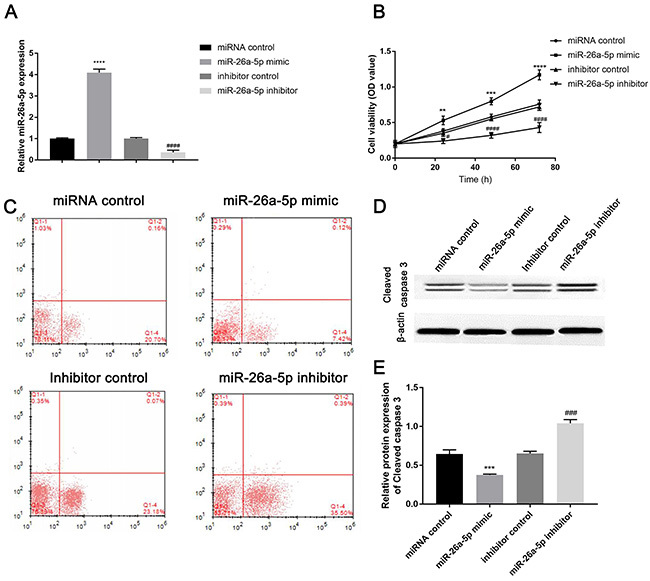
Effect of miR-26a-5p on cell viability and apoptosis. **A**, After cardiomyocytes were transfected with miR-26a-5p mimic, miRNA control, miR-26a-5p inhibitor, or inhibitor control and treated with hypoxia/reoxygenation, the expression of miR-26a-5p was evaluated by qRT-PCR. The (**B**) viability and (**C**) apoptosis of the cells were detected by MTT and flow cytometry, respectively. **D**, Cleaved caspase 3 expression was assessed by western blot and quantified by ImageJ software (**E**). Data are reported as means±SD. **P<0.01, ***P<0.001, ****P<0.0001 *vs* miRNA control; ^#^P<0.05, ^###^P<0.001, ^####^P<0.0001 *vs* inhibitor control (ANOVA).

miR-26a-5p level was greatly increased by miR-26a-5p mimic but decreased by miR-26a-5p inhibitor, which suggested that transfection was effective (Figure 3A, P<0.0001). Furthermore, MTT assay revealed that miR-26a-5p over-expression improved and enhanced the viability of cardiomyocytes, while miR-26a-5p knockdown markedly inhibited cell viability (Figure 3B). Hence, miR-26a-5p improved the viability of cardiomyocytes with I/R injury.

After transfection and H/R treatment in cardiomyocytes, a lower apoptotic rate of miR-26a-5p mimic group (7.54%) was observed compared to miRNA control group (20.86%), and yet miR-26a-5p inhibitor group had a higher apoptotic rate (35.89%) compared to the inhibitor control group (23.25%) (Figure 3C).

Furthermore, miR-26a-5p over-expression decreased the level of pro-apoptotic protein cleaved caspase 3, while knockdown of miR-26a-5p increased cleaved caspase 3 level compared to the control group (Figure 3D and E, P<0.0001). Collectively, miR-26a-5p could inhibit cardiomyocytes apoptosis induced by I/R injury.

### Interaction between miR-26a-5p and PTEN

**Figure 4:**
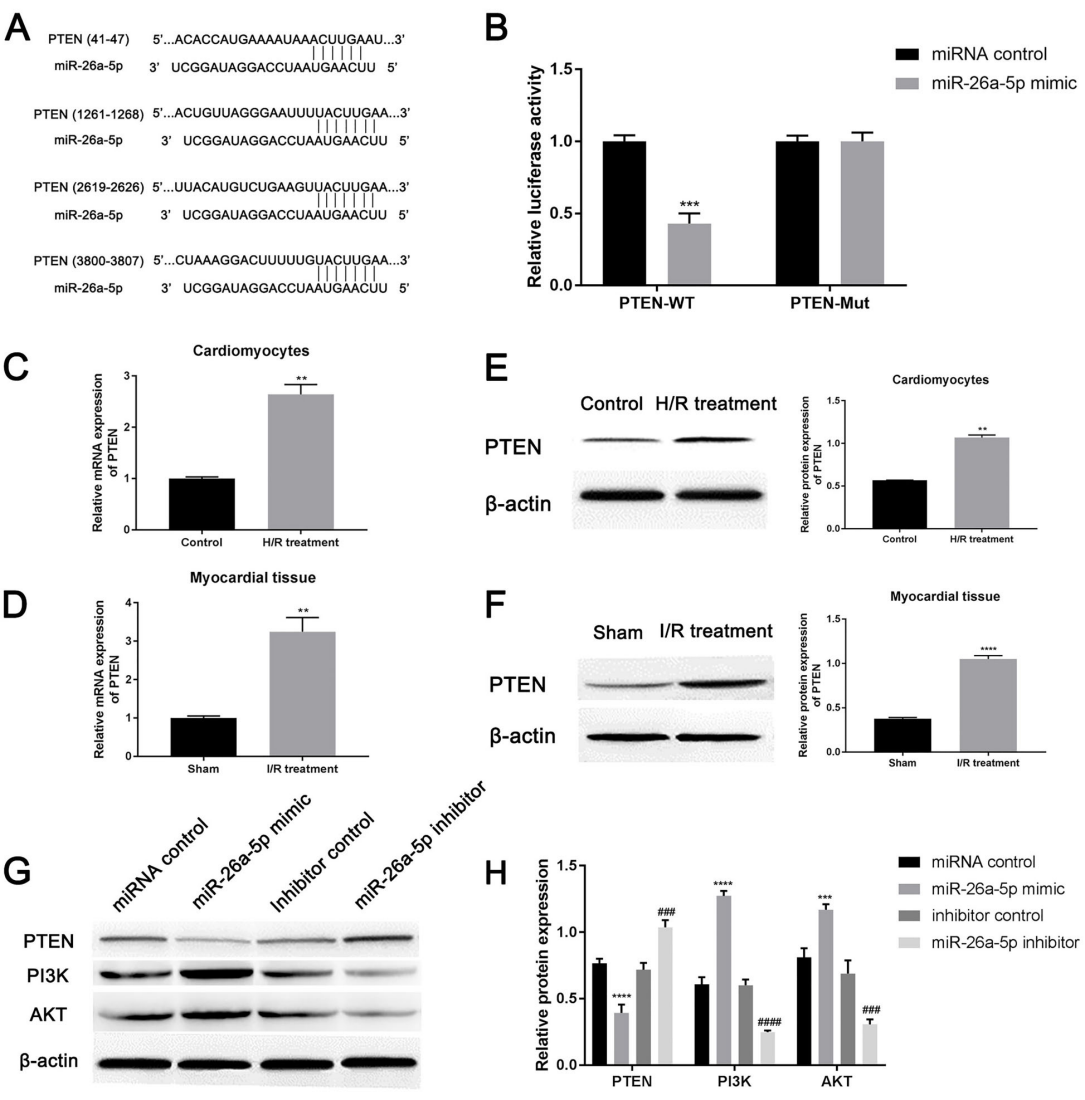
Interaction between miR-26a-5p and PTEN. **A**, Binding sites between miR-26a-5p and PTEN. **B**, Luciferase reporter assay measured the luciferase activity of PTEN-WT (wild type) or PTEN-Mut (mutant) vector. The mRNA and protein expression of PTEN in (**C** and **E**) cardiomyocytes after hypoxia/reoxygenation (H/R) and (**D** and **F**) myocardial tissue upon ischemia/reperfusion (I/R) injury was measured by qRT-PCR and western blot, respectively. After transfection of four different miR-26a-5p vectors, the expression of PTEN, PI3K, and AKT was evaluated by (**G**) western blot and quantified by (**H**) ImageJ software. Data are reported as means±SD. **P<0.01, ***P<0.001, ****P<0.0001 *vs* control groups; ^###^P<0.001, ^####^P<0.0001 *vs* inhibitor control (*t*-test or ANOVA).

PTEN was selected as a candidate, and four conserved binding sites of miR-26a-5p were observed in the 3′UTR of PTEN (Figure 4A). The relationship between PTEN and miR-26a-5p was further validated by luciferase reporter assay. Figure 4B shows that the luciferase activity of the PTEN-WT vector was obviously suppressed by miR-26a-5p compared to the control group (P=0.0003), while the activity of PTEN-MUT luciferase vector had no significant change between miR-26a-5p mimic transfection group and miRNA control transfection group (P>0.9999). Hence, PTEN was a direct target of miR-26a-5p.

After I/R injury treatment, the expression levels of PTEN in cardiomyocytes (Figure 4C, P=0.0038; Figure 4E, P=0.0011) and myocardial tissue (Figure 4D, P=0.0080; Figure 4F, P<0.0001) were up-regulated compared to the control groups. When cardiomyocytes were transfected with miR-26a-5p mimic, miRNA control, miR-26a-5p inhibitor, or inhibitor control, miR-26a-5p over-expression greatly decreased PTEN expression, whereas miR-26a-5p knockdown significantly increased PTEN expression compared to the control group (Figure 4G and H, P<0.0001). Thus, miR-26a-5p could negatively mediate PTEN expression.

Moreover, miR-26a-5p over-expression increased the expression of PI3K and AKT, and yet miR-26a-5p knockdown decreased the level of PI3K and AKT (Figure 4G and H, P<0.0001). As a plethora of studies suggest that PTEN is known as a negative regulator of the PI3K/AKT signaling pathway (22), we speculated that miR-26a-5p promoted the viability of H/R-induced cardiomyocytes and inhibited apoptosis by inhibiting PTEN expression to activate the PI3K/AKT signaling pathway.

## Discussion

Although reperfusion is currently the most effective treatment for AMI, it still causes a series of additional clinical damage (23). Thus, exploring the molecular mechanism is vital to conquer myocardial I/R injury (24). Like most miRNAs, miR-26a-5p functions in a diversity of biological processes such as cell apoptosis and metastasis (15,16). Additionally, up-regulation of miR-26a-5p in AMI ameliorates cardiac function and suppresses myocardial inflammation (17). miR-26a-5p can also inhibit the autophagy in cardiac fibroblasts through targeting ULK1, which is critical for cardiac hypertrophy and heart failure (18). Given the effect of miR-26a-5p in cardiovascular diseases, we attempted to study whether miR-26a-5p had an important role in myocardial I/R injury. Our study indicated that the expression of miR-26a-5p was significantly decreased in cardiomyocytes and myocardial tissue upon I/R injury compared to normal cardiomyocytes and myocardial tissue. Besides, the results in this study showed that up-regulation of miR-26a-5p significantly improved the viability of cardiomyocytes upon I/R injury, while down-regulation of miR-26a-5p obviously inhibited the cell viability. Moreover, miR-26a-5p over-expression greatly suppressed the cardiomyocyte apoptosis induced by I/R injury. Hence, miR-26a-5p could protect cardiomyocytes from I/R injury.

MicroRNAs are key actors in post-transcriptional regulation through regulating the expression of their mRNAs (25). At present, miR-26a-5p has been proven to control its gene targets and to be involved in cancer biology (15,16). For instance, miR-26a-5p facilitates the metastasis of lung cancer cells through targeting ITGβ8 (26). However, the mechanism of miR-26a-5p target regulation in AMI is not yet reported. Thus, the TargetScan website was used in this study to find the gene targets of miR-26a-5p, and PTEN was selected as a potential candidate. Compared with normal cardiomyocytes and myocardial tissue, our study proved that PTEN was over-expressed in cardiomyocytes and myocardial tissue upon I/R injury. Moreover, PTEN was observed to be negatively regulated by miR-26a-5p. PTEN, also named as MMAC1 or TEP1, is known as a tumor suppressor (27). PTEN can oppose the activation of the PI3K/AKT signaling pathway that plays an important role in cell survival, growth, metastasis, and metabolism (28). Therefore, we speculated that miR-26a-5p improved viability and suppressed apoptosis in cardiomyocytes upon I/R injury through inhibiting PTEN expression to activate the PI3K/AKT signaling pathway.

In conclusion, miR-26a-5p could effectively protect against myocardial I/R injury through controlling the PTEN/PI3K/AKT signaling pathway. The relationship among miR-26a-5p, PTEN, and PI3K/AKT pathway may offer a new insight for understanding the molecular mechanism of myocardial I/R injury, and provide a potential approach for treatment. However, this was a preliminary study and the pathogenesis of myocardial I/R injury is complex. Thus, large-scale studies will be needed to confirm the detailed mechanisms of miR-26a-5p, PTEN, and PI3K/AKT pathway in myocardial I/R injury through animal experiments and clinical tests in the future.
