# Erratum for: miR-26a-5p protects against myocardial ischemia/reperfusion injury by regulating the PTEN/PI3K/AKT signaling pathway

**DOI:** 10.1590/1414-431X20209106erratum

**Published:** 2020-03-02

Xiaowei Xing https://orcid.org/0000-0001-6433-2942 ^1^, Shuang Guo https://orcid.org/0000-0003-2705-057X ^2^, Guanghao Zhang https://orcid.org/0000-0002-3298-3211 ^1^, Yusheng Liu https://orcid.org/0000-0003-2317-6546 ^1^, Shaojie Bi https://orcid.org/0000-0002-9182-9855 ^1^, Xin Wang https://orcid.org/0000-0003-1122-5515 ^1*^, Qinghua Lu https://orcid.org/0000-0002-4450-3895 ^1*^

^1^Department of Cardiology, The Second Hospital of Shandong University, Jinan, Shandong, China

^2^Department of Gastroenterology, The Second Hospital of Shandong University, Jinan, Shandong, China

Correspondence: Qinghua Lu: <qinghualu@yeah.net>

^*^These authors contributed equally to this work.

**Erratum for:** Braz J Med Biol Res | doi: http://dx.doi.org/10.1590/1414-431X20199106

The authors notified the Editors of the Brazilian Journal of Medical and Biological Research that the internal controls of Figure 4E, 4F and 4G were incorrectly sent for publication. Thus, the corresponding semi-quantitative analyses of protein blots had to be recalculated and the histograms must be replaced. There were also errors in the text.

**Text corrections and revised Figure 1 and Figure 4.**

**Page 3,** left column, last line: The internal control was β-actin.

**Page 4,** Figure 1C and 1D. The internal control (Actin) has been changed to β-actin.

**Page 5,** right column, line 8 from bottom: Figure 4E, P=0.0001)

**Page 7,** last line of Figure 4 legend: ^###^P<0.001 should be deleted (as shown in the corrected legend and Figure 4 on page 3 of this erratum).

**Figure 1:**
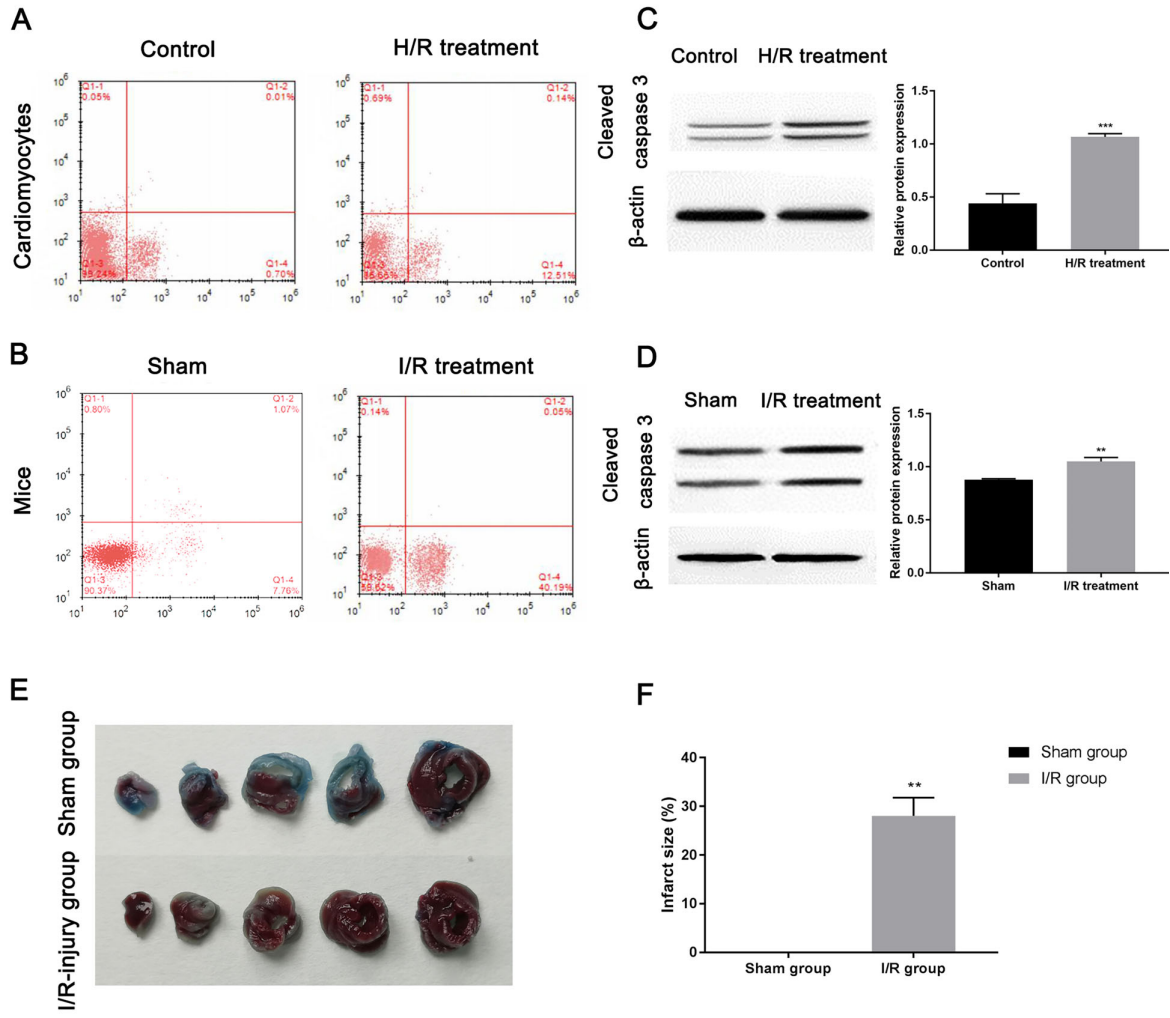
Establishment of ischemia/reperfusion (I/R) injury model. The images of flow cytometry show apoptosis in (**A**) cardiomyocytes and (**B**) myocardium of mice upon I/R injury. Western blot examined the expression of cleaved caspase 3 in (**C**) cardiomyocytes submitted to hypoxia/reoxygenation (H/R) treatment and (**D**) myocardial tissue upon I/R treatment. **E**, Representative images of Evans blue/TTC staining in five continuous slices of left ventricle from mice hearts treated with or without I/R treatment. **F**, The infarct size was quantified by Image-Pro Plus software. Data are reported as means±SD. **P<0.01, ***P<0.001 *vs* control groups (*t*-test).

**Figure 4:**
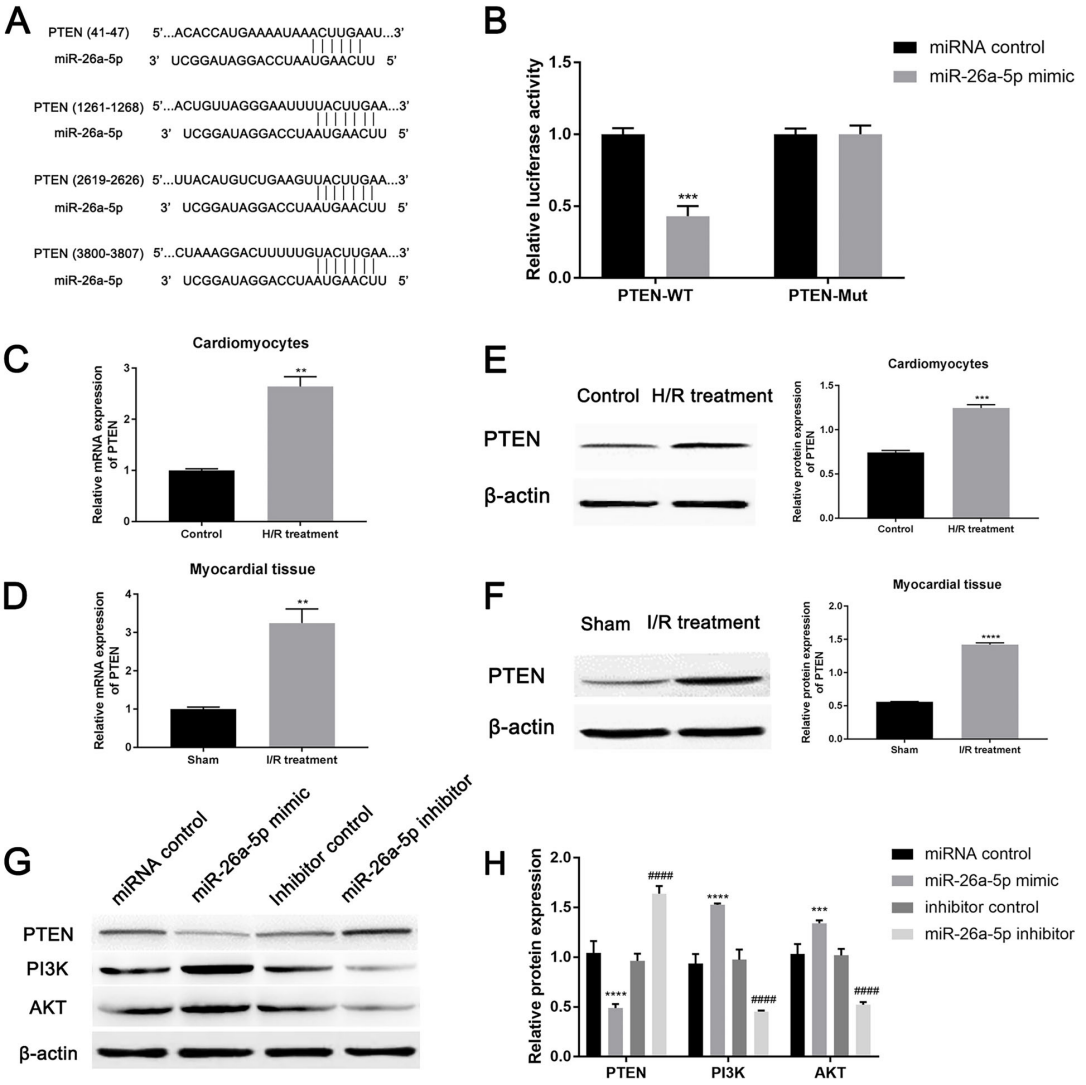
Interaction between miR-26a-5p and PTEN. **A**, Binding sites between miR-26a-5p and PTEN. **B**, Luciferase reporter assay measured the luciferase activity of PTEN-WT (wild type) or PTEN-Mut (mutant) vector. The mRNA and protein expression of PTEN in (**C** and **E**) cardiomyocytes after hypoxia/reoxygenation (H/R) and (**D** and **F**) myocardial tissue upon ischemia/reperfusion (I/R) injury was measured by qRT-PCR and western blot, respectively. After transfection of four different miR-26a-5p vectors, the expression of PTEN, PI3K, and AKT was evaluated by (**G**) western blot and quantified by (**H**) ImageJ software. Data are reported as means±SD. **P<0.01, ***P<0.001, ****P<0.0001 *vs* control groups; ^####^P<0.0001 *vs* inhibitor control (*t*-test or ANOVA).

